# Effects of Universal Adhesive on Shear Bond Strength of Resin Cement to Zirconia Ceramic with Different Surface Treatments

**DOI:** 10.1155/2021/5517382

**Published:** 2021-06-17

**Authors:** Zohreh Moradi, Farnoosh Akbari, Sara Valizadeh

**Affiliations:** ^1^Department of Restorative Dentistry, School of Dentistry, Tehran University of Medical Sciences, Tehran, Iran; ^2^Tehran University of Medical Sciences, Tehran, Iran; ^3^Dental Research Center, Dentistry Research Institute, Restorative Dentistry Department, School of Dentistry, Tehran University of Medical Sciences, Tehran, Iran

## Abstract

**Aim:**

This study aimed to assess shear bond strength (SBS) of resin cement to zirconia ceramic with different surface treatments by using Single Bond Universal.

**Methods:**

In this in vitro study, 50 zirconia discs (2 × 6 mm) were divided into 5 groups of (I) sandblasting with silica-coated alumina (CoJet)  + silane + Single Bond 2, (II) sandblasting with CoJet + Single Bond Universal, (III) sandblasting with alumina + Single Bond Universal, (IV) sandblasting with alumina + Z-Prime Plus, and (V) Single Bond Universal with no surface treatment. Resin cement was applied in plastic tubes (3 × 5 mm^2^), and after 10,000 thermal cycles, the SBS was measured by a universal testing machine. The mode of failure was determined under a stereomicroscope at × 40 magnification. Data were analyzed using one-way ANOVA.

**Results:**

The maximum (6.56 ± 4.29 MPa) and minimum (1.94 ± 1.96 MPa) SBS values were noted in groups III and I, respectively. Group III had the highest frequency of mixed failure (60%). Group V had the maximum frequency of adhesive failure (100%).

**Conclusion:**

Single Bond Universal + sandblasting with alumina or silica-coated alumina particles is an acceptable method to provide a strong SBS between resin cement and zirconia.

## 1. Introduction

Due to the increased demand for cosmetic restorations, ceramic restorations have become increasingly popular in the recent years [1]. Ceramics can be divided into two groups of silica ceramics such as feldspathic, leucite-reinforced and lithium disilicate-reinforced ceramics, and nonsilica ceramics such as alumina and zirconia [2]. Zirconia is zirconium dioxide, which was first used in orthopedics. Zirconia has gained growing popularity for dental applications in the recent years due to its favorable optical and mechanical properties and excellent biocompatibility. It can thus serve as an alternative to metal-ceramic restorations. The bond strength of zirconia depends on surface roughness and type of bonding agent used. Chipping and debonding are the most commonly reported shortcomings of zirconia restorations [3]. Debonding may occur due to the inadequate preparation of the abutment tooth, inappropriate bonding agent or cement selection, or wrong application technique [4, 5]. Unlike silica ceramics, zirconia does not have a glass phase. Thus, it cannot be etched with hydrofluoric acid [3] and requires other surface treatments such as preparation with diamond burs, sandblasting with aluminum oxide and silica-coated alumina particles (CoJet), Nd:YAG, Er:YAG, or CO_2_ laser irradiation, or a combination of these methods [6, 7].

For the bonding to zirconia, chemical adhesives are also required in addition to surface preparation, such as silane, and phosphate-containing primers or adhesives such as 10-methacryloyloxydecyl dihydrogen phosphate (10-MDP), 4-methacryloxyethyl trimellitate anhydride, and thiophosphoric methacrylate. They can cause chemical reactions with zirconia oxides and enhance the bond strength as such [8, 9]. Resin cements are the best adhesives for cementation of zirconia restorations and achieving optimal marginal adaptation, retention, and fracture resistance [10].

Use of different bonding agents with several application steps often confuse the clinicians, highlighting the need for simplification of the procedural steps, enhancing the bonding durability, and decreasing the technical sensitivity of the bonding procedure [11]. At present, universal adhesives are available in the market, which can bond to different types of metals and ceramics according to the manufacturers' claims. Also, they can optimally bond to dentin and enamel and can be used in self-etch, etch-and-rinse, and selective-etch modes [11].

Amaral et al. [11] evaluated the shear bond strength (SBS) of Scotchbond Universal to zirconia and reported that the SBS of this universal adhesive was higher than that of Z-Prime Plus, AZ Primer, and Monobond Plus. Sharafeddin and Shoale [10] compared the SBS of All Bond Universal with the conventional bonding agents to zirconia and reported that Z-Prime Plus along with sandblasting yielded higher SBS than the universal adhesive.

Considering the controversy [12, 13] in the reported results regarding the SBS of universal adhesives to zirconia and the novelty of universal adhesives, further studies are warranted on this topic. Thus, the purpose of this study is to compare the SBS of Single Bond Universal to zirconia ceramic with different surface treatments in comparison with Single Bond 2 and Z-Prime Plus.

## 2. Materials and Methods

### 2.1. Preparation of Zirconia Specimens and Surface Treatments

In this in vitro, experimental study, 50 zirconia discs measuring 6 × 2 mm^2^ were fabricated. For this purpose, presintered zirconia ceramics (CAD/CAM, Incoris Sirona, Hanau, Hesse, Germany) were sectioned by the Cercon system (Degudent, Dentsply International Company, Hanau, Hesse, Germany). All specimens were then sintered at 1550°C and polished with 600-grit carbide paper. The specimens were then randomly divided into 5 groups (*n* = 10) as follows:

  Group I: ten discs were sandblasted with 30 *µ*m silica-coated alumina particles (CoJet; 3M ESPE, St. Paul, MN, USA) under 2 bar pressure at 10 mm distance for 15 s using an intraoral sandblaster (Bio Art, Barcelona, Spain). The nonsandblasted surface of specimens was marked in all specimens. Next, silane (Bisco Inc., Schaumburg, IL, USA) was applied on the surface, allowed 1 min, and dried with air spray for 10 s such that its surface was no longer shiny. Then, Single Bond 2 (3M ESPE, St. Paul, MN, USA) was applied by a microbrush and after 15–20 s, it was dried with gentle air spray for 5 s. The specimens were then light-cured (Woodpecker, Henan, China) with 450 nm wavelength and 1000 mW/cm^2^ energy density (measured by a radiometer) for 10 s.
  Group II: ten discs were sandblasted with 30 *µ*m silica-coated alumina particles (CoJet; 3M ESPE, St. Paul, MN, USA) under 2 bar pressure at 10 mm distance for 15 s using an intraoral sandblaster (Bio Art, Barcelona, Spain). Single Bond Universal (3M ESPE, St. Paul, MN, USA) was then applied by a microbrush and after 15–20 s, it was dried with gentle air spray for 5 s. Another coat of bonding agent was applied on the surface and slightly dried by 5 s of gentle air spray. It was then light-cured (Woodpecker, Henan, China) with 450 nm wavelength and 1000 mW/cm^2^ energy density for 10 s.
  Group III: ten specimens were sandblasted with 50 *µ*m alumina particles (AL2O3, Henry west, Germany) under 2 bar pressure at 10 mm distance for 10 s using a laboratory sandblaster (Mestra, Germany). Then, Single Bond Universal was applied as explained earlier.
  Group IV: ten specimens were sandblasted with 50 *µ*m alumina particles (AL2O3, Henry west, Germany) under 2 bar pressure at 10 mm distance for 10 s using a laboratory sandblaster (Mestra, Germany). Next, Z-Prime Plus (Bisco Inc., Schaumburg, IL, USA) was applied by a microbrush in two layers for 15 s. After applying each layer, it was dried with gentle air spray for 15 s.
  Group V: Single Bond Universal was applied on the surface of the remaining 10 specimens as in groups II and III. This group served as the control group and did not receive any surface treatment.

Next, plastic tubes measuring 5 mm in diameter and 3 mm in height were placed on the surface and Dou-Link (Bisco Inc., Schaumburg, IL, USA) was applied in two 1.5-mm-thick increments into each tube such that after applying each increment, it was light-cured with 450 nm wavelength and 1000 mW/cm^2^ energy density (measured by a radiometer) for 40 s from 1.5 mm distance.

### 2.2. Thermocycling

The groups of cemented zirconia specimens were separately wrapped in a sterile gauze, coded with a waterproof marker, and placed in a thermocycler (Vafaei Industrial TC-300, Iran). They underwent 10,000 thermal cycles between 5–55°C with a dwell time of 20 s and a transfer time of 10 s.

### 2.3. SBS Testing

The mounted specimens underwent SBS testing in a universal testing machine (ZwickZ010/TN2A, Ulm, Germany) with a crosshead speed of 1 mm/min.

### 2.4. Mode of Failure

The mode of failure was determined under a stereomicroscope (Nikon, Tokyo, Japan) at × 40 magnification as adhesive (at the bonding/ceramic or cement interface), cohesive (within the bonding), mixed (a combination of both), and cohesive substrate (within the ceramic or cement).

### 2.5. Statistical Analysis

Data were analyzed using SPSS version 24 via one-way ANOVA at 95% confidence interval, assuming normal distribution of data. Considering the significant result, pairwise comparisons of SBS values and modes of failure were carried out using the Games-Howell and chi-square tests. *P* < 0.05 was considered statistically significant.

## 3. Results

### 3.1. SBS Results

A number (8 out of 50) of specimens were debonded after thermocycling.  Table 1 shows the number of remaining specimens after thermocycling. The SBS of debonded specimens was considered zero in statistical tests (pretest failure).

**Figure 1:**
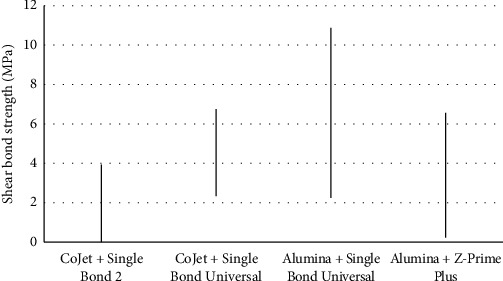
Mean and 95% confidence interval of shear bond strength of the study groups.

Table 2 shows the mean SBS of the five groups. The difference in SBS was significant among the five groups (*P*=0.008). Thus, pairwise comparisons were performed by the Games-Howell test, which revealed significant differences between groups I (1.96 ± 1.94 MPa) and II (4.54 ± 2.18 MPa) (*P*=0.03) and I (1.96 ± 1.94 MPa) and III (6.56 ± 4.29 MPa) (*P*=0.03). No significant differences were noted between groups I (1.96 ± 1.94 MPa) and IV (3.39 ± 3.14 MPa) (*P*=0.47), II (4.54 ± 2.18 MPa) and III (6.56 ± 4.29 MPa MPa) (*P*=0.56), II (4.54 ± 2.18 MPa) and IV (3.39 ± 3.14 MPa) (*P*=0.78), and III (6.56 ± 4.29 MPa) and IV (3.39 ± 3.14 MPa) (*P*=0.27).  Figure 1 shows the mean and standard deviation of SBS of the study groups.

### 3.2. Mode of Failure

Assessment of the mode of failure under a stereomicroscope revealed that the frequency of mixed failure was the highest (60%) in group III (alumina + Single Bond Universal). The maximum frequency of adhesive failure (100%) was noted in group V (Single Bond Universal) (Table 3).

**Figure 2:**
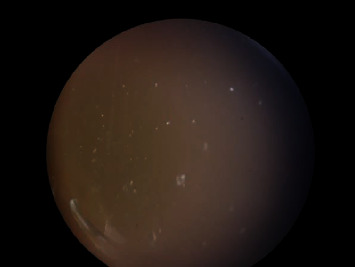
A specimen with adhesive failure under stereomicroscope (40x).

**Figure 3:**
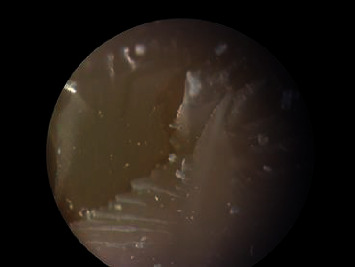
A specimen with mixed failure under stereomicroscope (40x).

Pairwise comparisons of the modes of failure by the chi-square test revealed a significant difference between groups III (alumina + Single Bond Universal) and V (Single Bond Universal) (*P*=0.02). No other significant differences were noted (*P* > 0.05). Stereomicroscope images of adhesive and mixed failures are shown in Figures 2 and 3, respectively.

## 4. Discussion

Considering the increasing demand for all ceramic restorations due to their favorable esthetics and high strength, search for a restoration with ideal physical properties is still ongoing. Also, a strong bond between the resin cement and zirconia is a challenge in dental treatments [14].

This study assessed the SBS of Single Bond Universal to ceramic with different surface treatments in comparison with Z-Prime Plus and Single Bond 2. The results showed that sandblasting with different particles, and in other words, mechanical preparation before the application of different bonding agents, significantly increased the SBS. In the control group (Single Bond Universal alone with no mechanical surface preparation), all specimens were debonded after 10,000 thermal cycles, which indicates low SBS of specimens without surface treatment. The results of previous studies regarding the efficacy of different zirconia surface treatments have been controversial. However, the main principle is the need for mechanical surface treatment before the application of bonding agent [15]. Sandblasting with different particles is believed to be the most efficient surface treatment according to a number of researchers such as Akyil et al. and Hosseini et al. [16, 17]. Sandblasting effectively increases the surface roughness of ceramics and also increases the available ceramic surface area [17]. Thus, sandblasting was used in this study for mechanical surface treatment of zirconia. Ozcan [18] performed sandblasting with 30–50 *µ*m alumina particles under 0.5–2.5 bar pressure from 10 mm distance for a maximum of 20 s to prevent damaging the zirconia surface. They suggested this protocol for sandblasting. The same protocol was adopted in this study. Erdem et al. [9] found no significant difference between sandblasting with alumina particles and CoJet and showed that they both increased the SBS. Their results were in line with our findings. In this study, the SBS was not significantly different in sandblasting with alumina and silica-coated alumina particles.

However, some studies have reported controversial results in this respect. Della Bona et al. [19] concluded that the tensile and shear bond strength after sandblasting with silica-coated alumina particles was higher than those after sandblasting with alumina particles. They explained that the zirconia surface coated with silica would provide a stronger bond to silane and resin [19]. Amaral et al. [11] reported that sandblasting with alumina yielded superior bond strength between resin cement and zirconia compared with sandblasting with silica-coated alumina. This result may be due to higher roughness or chemical interactions between the Al_2_O_3_ particles and primers, because primers containing MDP have affinity for metal oxides [11].

Amaral et al. and Ranjbar Omidi et al. used a universal adhesive and Z-Prime Plus, similar to our study, and reported that the universal adhesive along with sandblasting was an acceptable option for bonding of zirconia ceramics [11, 20]. Their results were in agreement with our findings. Universal adhesives have adhesive resin components in addition to MDP that allows easier flow of the cement. They are copolymerized along with resin cement and create a strong bond. Also, universal adhesives contain silane, which decreases the surface tension of the substrates. Thus, the surface energy increases and enhances suitable bonding. Therefore, it increases the bond strength of nonsilica ceramics such as zirconia and alumina [21].

However, the results of Sharafeddin and Shoale [10] were in contrast to our findings. They reported that Z-Prime Plus along with sandblasting yielded a higher bond strength than All Bond Universal. They explained that Z-Prime Plus includes MDP and carboxylic monomers, which can chemically interact with the zirconia oxide layer. It seems that the synergistic effect of MDP and carboxylic monomers is the most probable reason for higher bond strength in this group.

This study also showed that 10,000 thermal cycles caused debonding of some specimens. In the control group (Single Bond Universal alone), all specimens were debonded. However, debonding occurred in a small number of specimens in the sandblasting + bonding agent or primer groups, In the CoJet + Single Bond 2 group (group I), 5 specimens were debonded, while 3 specimens were debonded in the sandblasting with alumina + Z-Prime Plus group. No debonded specimen was noted in groups where Single Bond Universal was used after sandblasting, which indicates high bond strength and durability following surface roughening and application of Single Bond Universal.

In the study by Amaral et al. [11], the specimens underwent 2500 thermal cycles after preparation. After thermocycling, the maximum number of debonded specimens were noted in the groups that had not received mechanical surface treatment, which was similar to our results. They also reported that universal adhesive (despite debonding of some specimens in this group) could provide acceptable bond strength even without surface treatment. In our study, the control specimens (no surface treatment and use of universal adhesive alone) were all debonded after 10,000 thermal cycles. Difference between our results and those of Amaral et al. [11] can be due to the difference in the frequency of thermal cycles, since in their study, specimens underwent 2500 thermal cycles, while this value was 10,000 cycles in our study. It seems that lower number of cycles could not significantly affect the bond strength in their study. The instruction sheet of Single Bond Universal clearly states that sandblasting is preferred for mechanical preparation before the application of bonding agent; however, a control group was also considered in our study to assess the accuracy of this statement.

The results of Ozcan, Tsuo et al., Hallman et al., and Blatz et al. were also in line with our findings. They also reported that thermocycling significantly decreased the SBS of resin cement to zirconia [18, 22–24]. In their studies, the control group without mechanical surface treatment showed the maximum frequency of pretest failure, which indicates that increasing the micromechanical retention by sandblasting enhances the SBS. In general, according to the literature, sandblasting with alumina particles or CoJet along with the application of a bonding agent or primer-containing phosphate monomers creates a more durable bond compared with other methods. A low bond strength was noted in groups where universal bondings were used. Although sandblasting may enhance the bond strength, water sorption by Single Bond Universal, which contains a number of components, such as HEMA, 10-MDP, silane, dimethacrylate resins, filler, water, and ethanol, may decrease the bond strength after thermocycling [25].

The results of Al-Jaidi et al., Erdem et al., Chen and Shu, Atsu et al., andKumar et al. regarding the mode of failure were in line with our findings such that the majority or all failures in the control group were adhesive, whereas most failures were mixed in the sandblasted groups or when MDP-containing bonding agents were used. It seems that sandblasting before the application of bonding agent and use of primer or bonding agents containing MDP result in a strong bond between the zirconia and resin cement and consequently fewer adhesive failures [7, 9, 26–28].

## 5. Conclusion

Based on the results of this study and the limitation of this study, it was concluded that

  Mechanical surface treatment is imperative before the application of universal adhesives.
  The sole application of the universal bonding agent does not provide sufficient bond strength.
  The high number cycles of thermocycling makes this study closer to the real clinical condition.

## Figures and Tables

**Table 1:** Number of specimens before and after thermocycling.

Study groups	Before thermocycling	After 10,000 thermal cycles
Sandblasting with CoJet + Single Bond 2	10	5
Sandblasting with CoJet + Single Bond Universal	10	10
Sandblasting with alumina + Single Bond Universal	10	10
Sandblasting with alumina + Z-Prime Plus	10	7
Single Bond Universal	10	0

**Table 2:** Mean and SD of shear bond strength of the groups (MPa).

Groups	Mean ± SD
Sandblasting with CoJet + Single Bond 2	1.96 ± 1.94^a^
Sandblasting with CoJet + Single Bond Universal	4.54 ± 2.18^b^
Sandblasting with alumina + Single Bond Universal	6.56 ± 4.29^b^
Sandblasting with alumina + Z-Prime Plus	3.39 ± 3.14^ab^
Single Bond Universal	0.0 ± 0.0^c^

Similar lower-case letters indicate absence of a significant difference while dissimilar letters indicate a significant difference.

**Table 3:** Frequency percentage of modes of failure in the groups.

			Study groups				
			Sandblasting with CoJet + Single Bond 2	Sandblasting with CoJet + Single Bond Universal	Sandblasting with alumina + Single Bond Universal	Sandblasting with alumina + Z-Prime Plus	Single Bond Universal
Mode of failure	Adhesive	Number	8 (80%)	5 (50%)	4 (40%)	5 (50%)	10 (100%)
	Mixed	Number	2 (20%)	5 (50%)	6 (60%)	5 (50%)	0
	Cohesive	Number	0	0	0	0	0

## Data Availability

The data used to support the study are available from the corresponding author upon request.
